# Individual Differences in Male Rats in a Behavioral Test Battery: A Multivariate Statistical Approach

**DOI:** 10.3389/fnbeh.2017.00026

**Published:** 2017-02-17

**Authors:** Daniel D. Feyissa, Yogesh D. Aher, Ephrem Engidawork, Harald Höger, Gert Lubec, Volker Korz

**Affiliations:** ^1^Department of Pediatrics, Medical University of ViennaVienna, Austria; ^2^School of Pharmacy, College of Health Sciences, Addis Ababa UniversityAddis Ababa, Ethiopia; ^3^Core Unit of Biomedical Research, Division of Laboratory Animal Science and Genetics, Medical University of ViennaHimberg, Austria; ^4^Department of Pharmaceutical Chemistry, University of ViennaVienna, Austria

**Keywords:** individuality, principle component analysis, cognition, test battery, mood

## Abstract

Animal models for anxiety, depressive-like and cognitive diseases or aging often involve testing of subjects in behavioral test batteries. The large number of test variables with different mean variations and within and between test correlations often constitute a significant problem in determining essential variables to assess behavioral patterns and their variation in individual animals as well as appropriate statistical treatment. Therefore, we applied a multivariate approach (principal component analysis) to analyse the behavioral data of 162 male adult Sprague-Dawley rats that underwent a behavioral test battery including commonly used tests for spatial learning and memory (holeboard) and different behavioral patterns (open field, elevated plus maze, forced swim test) as well as for motor abilities (Rota rod). The high dimensional behavioral results were reduced to fewer components associated with spatial cognition, general activity, anxiety-, and depression-like behavior and motor ability. The loading scores of individual rats on these different components allow an assessment and the distribution of individual features in a population of animals. The reduced number of components can be used also for statistical calculations like appropriate sample sizes for valid discriminations between experimental groups, which otherwise have to be done on each variable. Because the animals were intact, untreated and experimentally naïve the results reflect trait patterns of behavior and thus individuality. The distribution of animals with high or low levels of anxiety, depressive-like behavior, general activity and cognitive features in a local population provides information of the probability of their appeareance in experimental samples and thus may help to avoid biases. However, such an analysis initially requires a large cohort of animals in order to gain a valid assessment.

## Introduction

Behavioral tests are common in various fields of basic and preclinical research such as pharmacology i.e., to test drug effects upon learning and memory and behavioral patterns. For this reason a variety of specific tests have been developed to assess the degree of anxiety- or depression-like behavior as well as cognitive abilities. In practice relatively small sample sizes of experimental animals are compared, i.e., in pharmacological studies. Furthermore, the animals often undergo a battery of tests in order to gain comprehensive results for different behavioral patterns. The results of these tests that indicate different behavioral dimension should be proved not to be highly correlated (Steptoe, 2010). However, intertask correlations in test batteries have been repeatedly found (Arendash and King, 2002; Learmonth et al., 2015). The principal component anaylsis with orthogonal rotation provides indepence of the factors, and the behavioral variables with high factor specific loadings, from each other. Experimental rats are randomly assigned to different treatments and the statistics should allow conclusions about the population. For this reason it would be of interest to know how and to what extent these individual features are distributed in a population of intact untreated animals under laboratory conditions. In order to obtain a valid assessment the analysis of a large cohort of animals is required (Wall and Messier, 2001). Therefore, we tested a cohort of 162 adult male Sprague-Dawley rats in a battery of tests that are commonly used to assess cognitive abilities and behavioral patterns. We reduced the high number of variables to a lower number of representative factors by using a principal component analysis in order to extract components related to cognition, activity, anxiety-, and depression like behavior from the complex behavioral data. Factor analysis in the evaluation of behavioral data has previously been used to explore relations between tests (File, 1993), sex specific behavior (Carreira et al., 2017) or the validity of behavioral variables within a test paradigm (Crusio, 2001; Wall and Messier, 2001; Arantes et al., 2013; MacKillop et al., 2016), separating variables. Here, we report the effectiveness of this approach to assess individuality by considering the factor scores of individual animals, thus separating individuals. High or low loadings of individual scores on specific factors give information about the individual behavioral patterns in comparison to other individuals of the population. The aim of the study was to test the multivariate approach on its effectivity to extract meaningful components out of the high complexity of the data and whether the distribution of these individual features in a population of naive animals can be assessed. The study did not aim at developing a comprehensive test battery for evaluation of cognition and anxiety- and depression like behavior, this has to be done by researchers according to their individual needs. The answers may allow conclusions about the probability of having individuals with trait behavioral patterns (Wall and Messier, 2001), anxious- or depression related behavior in the test samples that were recruited for experimental or control animals, as well as for effects between different behavioral tests. The classification suggested here may contribute to improve sample validity for experiments, interlaboratory validity of experimental results and the characterization of individual animals.

## Methods

### Animals

One hundred and sixty two male Sprague Dawley rats, aged between 5 and 6 months, bred and maintained in standard Makrolon cages (length: 60 cm, width: 34, height 20 cm; 3 animals per cage) filled with autoclaved woodchips in the Core Unit of Biomedical Research, Division of Laboratory Animal Science and Genetics, Medical University of Vienna, were used. Food (sniff®, Soest; Germany) and tap water was available *ad libitum*. Facility conditions were: temperature: 22 ± 2°C; humidity: 55 ± 5%; 12 h artificial light/12 h dark cycle: light on at 7:00 a.m.). All procedures were carried out according to the guidelines of the Ethics committee, Medical University of Vienna, and were approved by Federal Ministry of Education, Science and Culture, Austria. The animals are subsequently used in another study, the present tests representing a pretest to that. The follow-up study requires this number of animals and at the same time allows multivariate statistics.

### Behavioral tests

Behavioral tests were performed in the following sequence: Elevated plus maze, open field, rota-rod, forced swim test, holeboard test. The apparatuses (Bilek+Schüll GMBH; Vienna, Austria) were custom made as well as the software (TIBE V.1.0; Vienna, Austria) for analysis. Hole visits in the holeboard were recorded manually from videos.

The between tests interval was 1 week. Because the ratio between the number of analyzed variables and number of individuals is critical for the validity of PCA results we chose one common test only for each feature. Also the number of variables within each test was minimized to those with the highest variance between individual rats.

These tests represent a standard test battery used for the evaluation of side effects in pharmacological studies (Sunyer et al., 2007, 2008).

### Elevated plus maze

The elevated plus maze is a common test to assess anxiety (Belzung and Griebel, 2001). It consists of a cross of two closed arms with side walls and two open arms. Rats and mice usually prefer to stay in closed arenas and avoid the exploration of open arenas (Carobrez and Bertoglio, 2005; Arantes et al., 2013). Thus, the longer the animals stay on open arms the lesser they are considered to be anxious. The elevated plus maze consists of two plus shaped plastic lanes (10 cm in width, 1.10 m in length at a height of 62 cm), with one lane (closed arm) surrounded by black plastic walls (40 cm in height), except at the crossing point with a gap of 10 cm, so that each arm had a length of 50 cm. The animals were placed in this center and allowed to explore the maze for 5 min. The movements were recorded by a tracking system and stored on a computer. The ratio of the time (s) the animals spent in the closed and the open arms (EPMRCO), the ratio of distances traveled and the rest in closed and open arms (EPMRDOC), the local movement (EPMlocal, <10 cm/s) and the number of entries into the open arms were considered for the analysis. The last three variables were choosen in order to compare it with corresponding variables in the open field (Carola et al., 2002) with regard to locomotion and the motivation to explore open arenas (the exploration of the central arena in the open field was almost invariant between animals).

### Open field

The open field test is widely used to test exploratory behavior and general activity of mice and rats (Crusio, 2001). The open field consists of a black plastic board (1.20 m × 1.20 m) surrounded by black plastic walls (50 cm in height). The rats were placed in the arena and allowed to explore it for 10 min. The movements were recorded by a video tracking system and stored on a computer. Considered were the ration between distance traveled (m), and the time the animals did not move (s) OFRDR, local movements (OFlocal, <10 cm/s) and the average velocity of movement (OFveloc; m/s). The usually used variables of the ration of the time spent in the central and peripheral part of the arena in order to assess the motivation to voluntarily explore unsafe areas were not considered because there was minimal variability between animals, thus they do not provide information about individuality and unnecessarily increases the number of variables in the PCA. In order to provide information about the readiness to enter unsafe areas the number of entries in open arms of the elevated plus maze test has been considered (Carola et al., 2002).

### Rota rod

The rota-rod is a widely used test to assess motor disabilities of rodents. The time-length the animals are able to stay on a rotating rod is considered to be a correlate of motor abilities (Brooks and Dunnett, 2009). The apparatus consists of four adjacent rods (10 cm in width at a height of 40 cm) separated with plastic barriers. The animals were placed on the rods which rotated with increasing speed. Rats were tested three times. The time until the rats fall off the rods was measured and the mean time (RRtime; s) for the three tests was used for analysis.

### Forced swim test

The forced swim test is a common test to assess depressive-like behavior. Therefore the time the animals are immobile and not struggling to escape from the water is considered to be a correlate of depressive-like behavior (Slattery and Cryan, 2012). The test consists of a training and a test session on two consecutive days. The rats were placed in a translucent plastic cylinder (20 cm in diameter and 45 cm in height filled with water up to a 33 cm level at a temperature of 25°C). The rats were placed in the cylinder for 10 min during the training and 5 min during the test session. Movements in the cylinder were recorded by a video tracking system and stored on a computer. The time (s) the animals spent immobile during training (FSIMTR) and during test (FSIMT) were used for the analysis.

### Holeboard

The holeboard is a test of spatial learning and memory and, by using the same protocol as in the present study, has been found to be reliable and highly sensitive to pharmacological interventions, induction, and maintenance of neuronal plasticity, and discrimination between physiological states of animals (Uzakov et al., 2005; Korz and Frey, 2007; Schulz and Korz, 2010; Meyer and Korz, 2013). The apparatus consists of a black plastic board (1 m × 1 m) with 16 regularily arranged holes (7 cm in diameter and 7 cm in depth). The board is surrounded by translucent plexiglass walls (30 cm in height). Outside at each wall black and white figures (A4) were mounted as proximal cues (Meyer and Korz, 2013). Distal cues consisted of further figures (A3) at room walls, furniture and equipment. Below the board a second board was placed densely scattered with the reward food pellets (dustless precision pellets of 45 mg; Bioserv®, Somerville, NJ, USA) to avoid possible olfactory orientation. During training four holes were baited. Trials were monitored via a video camera and stored on a computer. During the test procedure the animals were food deprived to reach 85% of their initial body weight (which is the widely used procedure) and stayed in the experimental room throughout the whole testing period. The experimental procedure was as follows: 3 days of weighing and handling (15 min) with providing 15–20 reward pellets at day 3 in the housing cage in order to familiarize the animals with the pellets. Two days of habituation (15 min) during which the animals were allowed to explore the maze with reward pellets in each hole and on the surface of the board. Three days of training and testing with 5 trials at day 1, four trials at day 2, and one retention trial at day 3. The trials ended after 2 min or when rats have found all 4 pellets. After each trial the board was cleaned with 0.1% incidin® solution to remove olfactory marks, feces, and urine. Counted were the reference memory errors (visits of unbaited holes) and visits and revisits of baited holes. A reference memory index (RMI) was calculated as: total visits of baited holes/total visits of all holes; thus a value of 1 indicates a good reference memory without errors) and the values for trial 1, 6, and 10 (HB-trial 1, 6, 10; respectively) were considered for the factor analysis.

### Statistics

Each variable was separately analyzed for minimum and maximum values, median, 25 and 75% percentiles, arithmetic mean, standard deviation, and standard error, the lower and the upper 95% confidence interval. Correlations between the measures of each test were detected by the Pearson product moment correlation (two-tailed). In order to reduce the number of dimensions a Factor analysis (principal component with Varimax rotation and Kaiser normalization, maximally 25 iterations to convergence) based on the correlation matrix was perfomed for all variables. Factor rotations simplify the interpretation of the factors. The coordinate axes rotation allows a better distribution of the loadings on the factors. The varimax rotation has been choosen because it is a orthogonal rotation method so that the different factors do not intercorrelate, thus each factor represents an independent behavioral pattern. Varimax rotation reduces the number of variables with high multiple factor loadings. Extracted components with eigenvalues higher than one were considered for further interpretation. In order to separate the animals according to their individual factor loadings (−1 to 1) on the components, loadings lower than −0.7 and higher than 0.7 were considered as border. Factor and correlation analyses have been done with SPSS Version 20, all other analyses by using GraphPad Prism (Version 5.02).

## Results

### General measures

Almost all behavioral variables were not Gaussian-distributed (Shapiro-Wilk normality test): HB-trial 1, *W* = 0.82, *p* < 0.001; HB-trial 6, *W* = 0.67, *p* < 0.001; HB-trial 10, *W* = 0.70, *p* < 0.001; OFRDR, *W* = 0.86, *p* < 0.001; OFveloc, *W* = 0.81, *p* < 0.001; RR-time, *W* = 0.97, *p* < 0.001; FSITR, *W* = 0.92, *p* < 0.001; FSIT, *W* = 0.56, *p* < 0.001; EPMRCO, *W* = 0.33, *p* < 0.001; EPMlocal, *W* = 0.81, *p* < 0.001; EPMRDOC, *W* = 0.61, *p* < 0.001. Normally distributed are the variables OFlocal, *W* = 0.98, *p* = 0.066 and EPMOE, *W* = 0.98, *p* = 0.071. Minimum and maximum values, percentiles, arithmetic means, standard deviation and error as well as confidence intervals are given in Supplementary Table 1. The different numbers of values results from some missing values for the different tests. The relative distribution of the raw data are given in Supplementary Figure 1.

### Linear regression

The results of the linear regression analysis by using the Pearson coefficient and the resulting *p*-values are given in Table 1. All of the open field variables are positively correlated with those of the late (trial 6 and trial 10) holeboard memory indices. Local movements in the open field are also positively correlated with the RMI of holeboard trial 1. EPMlocal positively correlates with FSIMT and there is a larger number of correlations of the number of open arm entries, positively with HBtrial6 and with movement parameters in the open field. Different measures within the tests were necessarily correlated and given here for reasons of completeness.

**Table 1:** **Correlationmatrix (Coefficient r after Pearson), and ***p***-value (two-tailed) of the between and within correlations of the different variables**.

	**HB-trial1**	**HB-trial6**	**HB-trial10**	**OFRDR**	**OFlocal**	**OFveloc**	**RRtime**	**FSIMT**	**FSIMTR**	**EPMRCO**	**EPMlocal**	**EPMOE**
HB-trial 6	**0.50**											
*p*	**<0.01**											
HB-trial10	**0.43**	**0.73**										
*p*	**<0.01**	**<0.01**										
OFRDR	0.13	**0.18**	**0.18**									
*p*	0.10	**0.02**	**0.02**									
OFlocal	**0.17**	**0.17**	**0.17**	**0.92**								
*p*	**0.03**	**0.03**	**0.03**	**<0.01**								
OFveloc	0.11	**0.18**	**0.17**	**0.92**	**0.88**							
*p*	0.17	**0.02**	**0.03**	**<0.01**	**<0.01**							
RR-time	−0.07	0.08	0.00	−0.71	−0.13	−0.10						
*p*	0.36	0.34	0.98	0.39	0.10	0.25						
FSIMT	−0.01	−0.05	−0.08	−0.04	−0.03	−0.04	−0.09					
*p*	0.88	0.49	0.33	0.60	0.69	0.59	0.29					
FSIMTR	0.05	−0.01	−0.05	−0.08	−0.07	−0.12	−0.09	**0.35**				
*p*	0.56	0.93	0.52	0.34	0.34	0.14	0.26	**<0.01**				
EPMRCO	−0.05	−0.08	−0.06	−0.05	−0.03	−0.05	0.00	0.00	−0.04			
*p*	0.48	0.31	0.47	0.50	0.67	0.49	1.00	0.99	0.63			
EPMlocal	−0.01	−0.14	−0.12	−0.33	0.08	−0.10	−0.06	**0.28**	−0.02	**0.18**		
*p*	0.92	0.08	0.12	0.68	0.34	0.20	0.45	**<0.01**	0.79	**0.02**		
EPMOE	0.05	**0.17**	0.15	**0.26**	**0.28**	**0.26**	−0.08	0.08	0.05	−**0.31**	−0.01	
*P*	0.49	**0.03**	0.06	**<0.01**	**<0.01**	**<0.01**	0.34	0.33	0.54	**<0.01**	0.87	
EPMRDOC	−0.02	−0.07	−0.05	−0.06	−0.04	−0.06	−0.01	0.02	−0.02	**0.93**	**0.17**	−**0.29**
*P*	0.78	0.35	0.56	0.42	0.60	0.48	0.93	0.78	0.84	**<0.01**	**0.03**	**<0.01**

*Significant correlation coefficients and the corresponding significance level (p) are given in bold. HB-trial 1, 6, 10: Holeboard reference memory index for trial 1, 6, and 10; respectively. OFRDR, -local, -veloc: Ratio between distance traveled and resting, local movement and mean velocity in the open field; respectively. RR-time: time to be on the rotarod. FSIMTR, -T: time in percent spent immobile in the forced swim task during training and test session, respectively. EPMRCO, -local, -OE, -RDOC: Ratio between time spent in open and closed arms, local movement, number of entries in open arms, ratio between distance traveled in open and closed arms on the elevated plus-maze; respectively*.

### Factor analysis (principal component)

**Figure 1:**
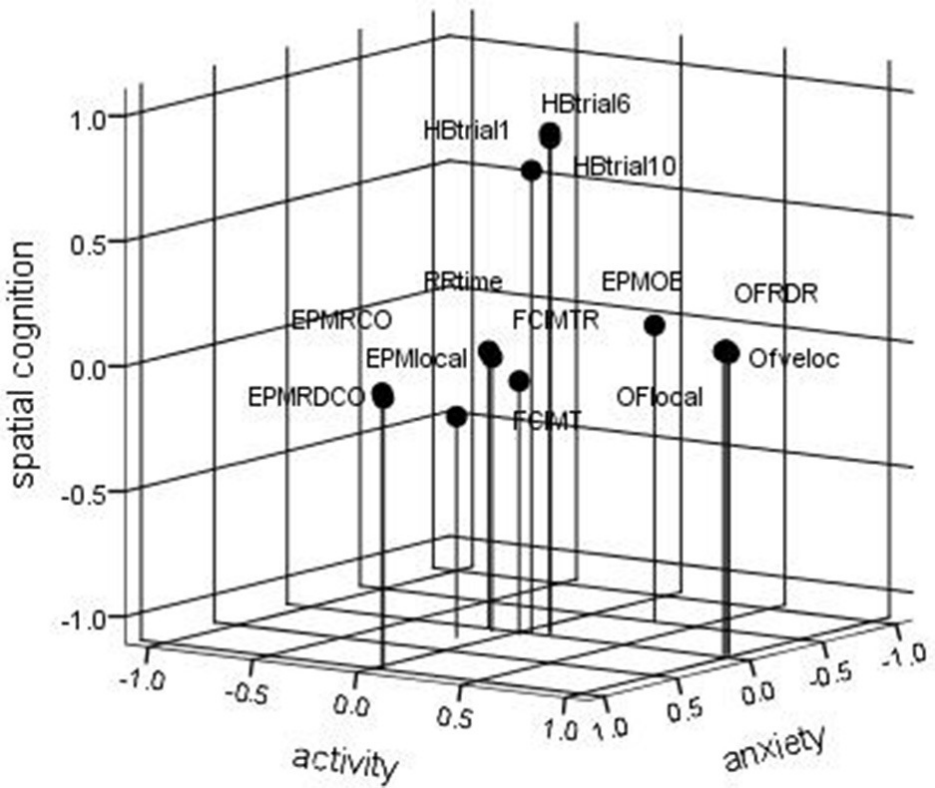
**Diagram of the first three components in rotated space**. HB-trial 1, 6, 10: Holeboard reference memory index for trial 1, 6, and 10; respectively. OFRDR, -local, -veloc: Ratio between distance traveled and resting, local movement, and mean velocity in the open field; respectively. RR-time: time to be on the rotarod. FSIMTR, -T: time in percent spent immobile in the forced swim task during training and test session, respectively. EPMRCO, -local, -OE, -RDOC: Ratio between time spent in open and closed arms, local movement, number of entries in open arms, ratio between distance traveled in open and closed arms on the elevated plus-maze; respectively.

**Figure 2:**
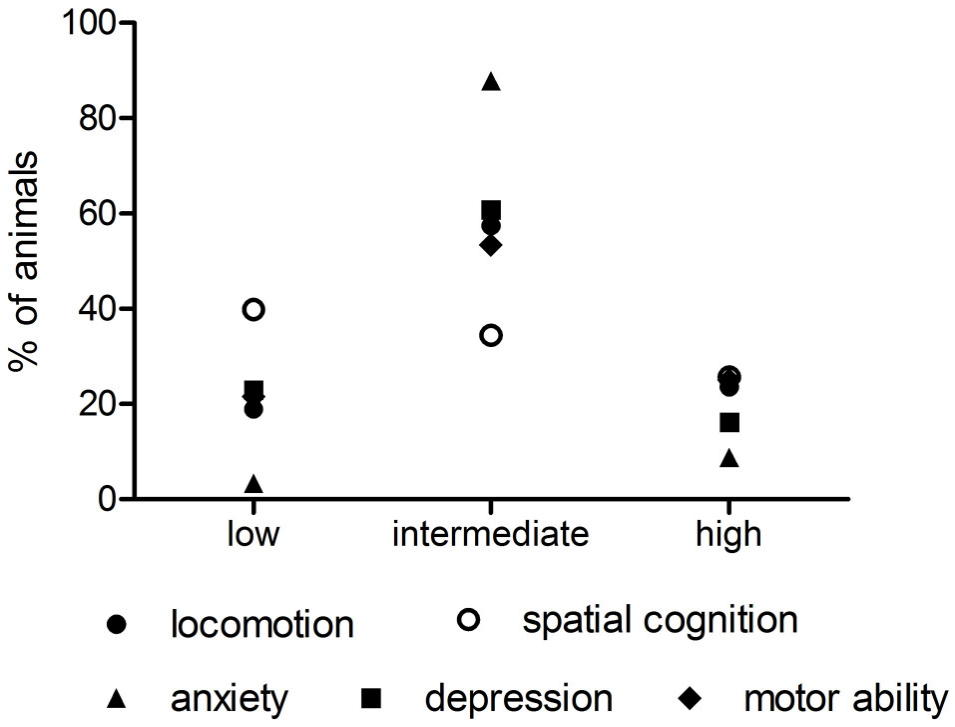
**Distribution of individual rats with high, low or intermediate levels of activity, spatial cognition, anxiety, depression and motor ability in the population of rats**.

The main results of the principal component analysis are given in Table 2. Considered were only components with an eigenvalue higher than 1. The first three dimensions and the euclidean distances of the variables are given in Figure 1. The first component explains 22.3% of the total variance (after rotation) and is positively highly positively loaded by the open-field variables OFRDR, OFlocal, and OFveloc. All of these variables general acticity this component was considered as representing the trait behavioral pattern to be active. The second component, explaining 16.8% of the variance is loaded by the holeboard reference memory indices and therefore considered as the spatial cognition component. The third component explaining 16.7% represents anxiety as it is characterized by EPM measures EPMROC and EPMRDCO the classic test for anxiety-like behavior. The number of entries in open arms is negatively loaded on this component, however does not reach the criterion of 0.7 factor score. Component 4 reflects depression-like behavior since the results of the test for depression, the forced swim immobility during test loads positively on these factor. Interestingly, also the immobility time during training is highly loaded it does not reach the criterion, although this variable is highly correlated with FSIMT. For that reason a high positive loading of individal rat scores on this component reveals high depression-like behavior. The rotarod measure is highly loaded on factor 5 which is therefore classified as reflecting motor abilities. Factor scores of −0.7 and 0.7 are considered as significant, wheras all values between these borders are considered as intermediate, thus may represent “normal” behavioral patterns. Because the animals are naive and not treated (except the tests), these behaviors are considered as trait patterns (Crusio, 2001). Similar high positive (>0.7) or negative (< −0.7) loadings of individual rat scores on component 1 reveal high vs. low general activity, with the rest representing 'normal' activity within the population. By doing this also for the other components, a pattern of the distribution of spatial cognitive abilities, activity, anxiety-, and depression-like behavior and motor abilities for this population of rats can be calculated and is given in Figure 2. Thus, 23.6% show a high, 18.9% a low, and 57.4% a intermediate activity. The largest “low” (39.9%) and the highest “high” portions are given for the factor spatial cognition and the largest “intermediate” portion (87.8%) for the intrinsic anxiety. Activity, depression and motor ability are close together with regard to their distributions over the three categories, whereas spatial cognition and anxiety differ. Because these results are based on a large cohort of rats we can assume that this is a characteric feature for male Sprague-Dawley rats of this age and that an experimenter recruiting experimental samples can expect these probabilities of indiviudal behavior in experimental samples.

**Table 2:** **Rotated (Varimax-Kaiser normalization) matrix of extracted components with eigenvalues greater than 1**.

	**1 (22.3%)**	**2 (16.8%)**	**3 (16.7%)**	**4 (11.7%)**	**5 (8.4%)**
	**Activity**	**Spatial cognition**	**Anxiety**	**Depression**	**Motor abilities**
HB-trial1	0.051	**0.737**	−0.014	0.033	−0.242
HB-trial6	0.096	**0.883**	−0.076	0.005	0.149
HB-trial10	0.128	**0.871**	−0.031	−0.060	0.101
OFRDR	**0.963**	0.092	−0.065	−0.014	0.015
OFlocal	**0.949**	0.100	−0.049	−0.032	−0.086
OFveloc	**0.951**	0.103	−0.062	−0.040	−0.022
RR-time	−0.106	−0.018	0.033	−0.120	**0.878**
FSIMT	0.024	−0.100	0.031	**0.816**	−0.150
FSIMTR	−0.157	−0.006	−0.017	0.686	−0.231
EPMRCO	0.023	−0.035	**0.961**	−0.002	−0.002
EPMlocal	−0.139	−0.231	0.228	−0.591	−0.414
EPMOE	0.296	0.072	−0.507	0.124	−0.014
EPMRDCO	0.015	−0.016	**0.958**	0.027	−0.014

*Percent values give the portion of explained variance for each factor. Significant factor loadings (>0.7, < −0.7) are given in bold. HB-trial 1, 6, 10: Holeboard reference memory index for trial 1, 6 and 10; respectively. OFRDR, -local, -veloc: Ratio between distance traveled and resting, local movement and mean velocity in the open field; respectively. RR-time: time to be on the rotarod. FSIMTR, -T: time in percent spent immobile in the forced swim task during training and test session, respectively. EPMRCO, -local, -OE, -RDOC: Ratio between time spent in open and closed arms, local movement, number of entries in open arms, ratio between distance traveled in open and closed arms on the elevated plus-maze; respectively*.

**Figure 3:**
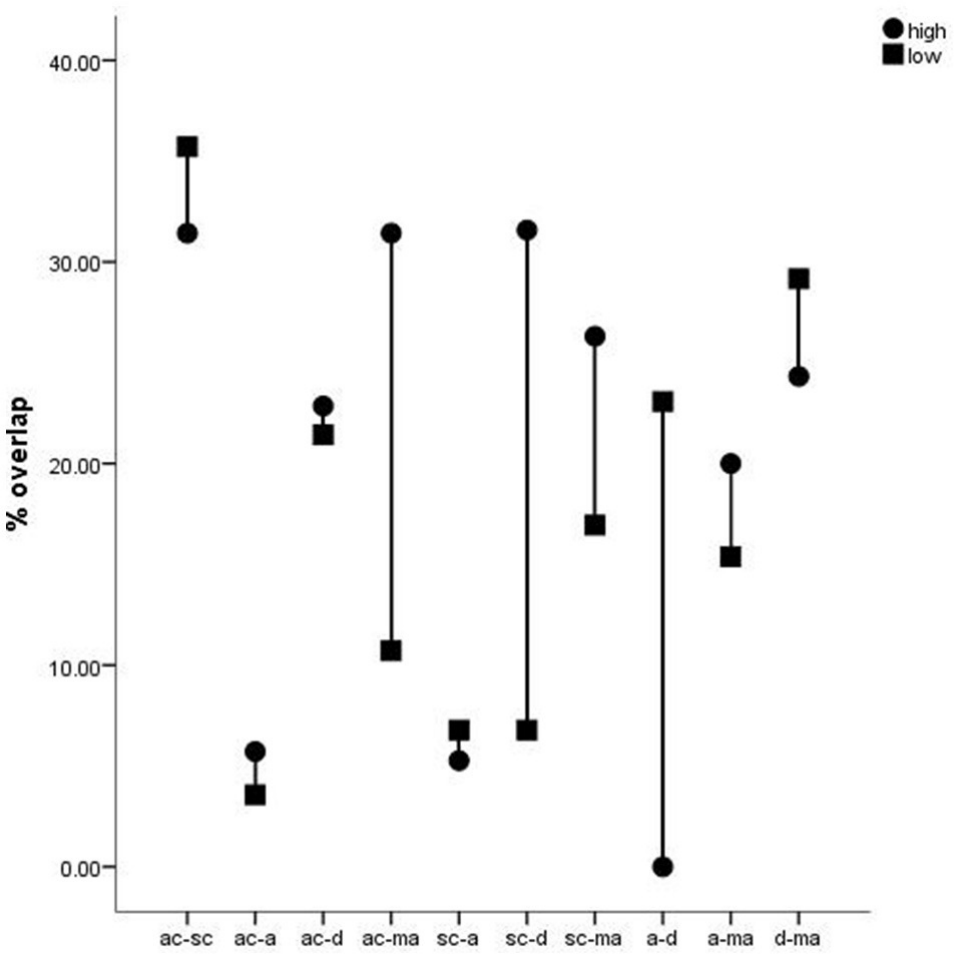
**Overlap (double appearance) of individual rats in high and low portions of factor loadings in percent**. ac-sc, -a, -d, -ma: activity and spatial cognition, anxiety, depression, motor ability, respectively. sc-a, -d, -ma: spatial cognition and anxiety, depression, motor ability, respectively. a-d, -ma: anxiety, motor ability, respectively. d-ma: depression and motor ability.

In order to reveal different high or low factor loadings in individual rats we counted double appearances in the different high and low portions of the factors. This overlaps (in %) are indicated in Figure 3. High percent values of individual rats are given for the factors activity and spatial cognition (ac-sc) and depression and motor abilitiy (d-ma) with little difference between high and low performers. The combinations ac-ma, sc-d, and a-d show the highest differences between high and low performers. There is no overlap at all between anxiety and depression in high performers in contrast to low performers, whereas a big overlap between spatial cognition and depression appears in the first.

## Discussion

We found discrete distributions of cognitive abilities and trait behavioral patterns in a large cohort of 5–6 months old male Sprague-Dawley rats. The lowest portion that exceed intermediate levels of behavior could be determined for anxiety, thus for experiments focusing on modulations of this behavior experience or pharmacological treatment the probability to include animals with an high or low trait background is the lowest. Cognition is most critical with a high portion of animals with low cognitive abilities, whereas that of high abilities is comparable to the other variables. This may partly be based on the age of the animals since often younger animals are used for experiments, which may be still more flexible and adaptive to task demands. However, only at this age male rats are fully adult in terms of social competence, brain development and musculoskeletal maturity (Adams and Boice, 1989; Sengupta, 2011; Mengler et al., 2014). The use of adult animals is of special interest because a lot of physiological and molecular phenomena change even over the first 3 months of age (McCutcheon and Marinelli, 2009).

The advantage of using PCA for the classification of individual animals is that a highly complex bundle of variables of different behavioral tests (but also other variables like molecular markers or physiological measures can be included) can be reduced to only a few components that reflect the behavioral features that are usually aimed to be assessed by these variables. Further, the most important indicators for specific behavioral patterns can be identified by the numerical factor scores and less important variables may be excluded in further studies (Wall and Messier, 2001). This on one hand may help to reduce the number of tests in a battery and on the other hand to improve the preconditions for sample size estimations. Power analysis that is done in order to determine the number of animals in an experimental sample to reach a reliable error limitation (to avoid false positive or negative statistical results), a specific power and effect size is affected by the variance of the analyzed data. The variance, however is different for variables between tests and even within tests, which has also been found in the present study. Further, power calculations have to be performed specifically for different statistical tests like linear regression or differences between means. Thus, simplifying preconditions for power calculations (i.e., by using factor scores) can reduce the number of calculations and probably also the number of animals used. The components extracted by the PCA are loaded significantly by behavioral variables of the different tests exclusively, making the assignment to underlying behaviroal patterns more easy. This is not the case in the linear regression analysis in which variables between tests are correlated. The disadvantage of the method is that initially a high number of animals have to be used since the validity of the results depends on the ratio of test variables and objects, which in many PCA studies is not optimal (Wall and Messier, 2001). The high percentage of overlap between activity and spatial cognition, between anxiety and depression in low performers and that between activity and motor ability in high performers may result from a mutual dependence beween these behaviors. The 30% overlap between spatial cognition and depression-like behavior in high performers, however is less clear since depression has often been described to result in lower cognitive abilities. However, this may be related to environmentally induced depression and not to an innate tendency to depression-like behavior.

The tests and the variables used have to be adapted to the needs of the experimenters, but may improve the communication between laboratories by identification of variables and tests that show similar loadings upon the extracted factors and not by comparing specific isolated variables.

The second focus was on comparing the PCA with the linear regression analysis results. We found correlations between the performance in different tasks, the open field variables OFRDR and velocity positively correlate with the the holeboard performance only at later but not the first trial, whereas local movements in the open field correlate with RMI at all trials. The similarity in shape and size between open field and holeboard and the associated familiarity with the arena may be one reason for these correlations. However, three habituation sessions preceded the holeboard test and habituation experience is similar for all rats, so that habituation as such should play a minor role. The enhanced activity is more likely to support holeboard learning and spatial memory. Further, most correlations could be observed when learning plays a major role, namely at the second day, and when a memory trace has been established at day 3 during the retention trial. The number of entries in open arms of the EPM, thus the readiness to explore unsafe arenas is higly positively correlated with the OFRDR and the OFlocal variables and weakly with RMI during the main learning phase in the holeboard. Immobility during forced swimming in the training and test sessions are also highly correlated suggesting that immobility during both sessions reflect the same level of depression-like behavior. However, in the PCA these performances could be separated such that only the test performance is significantly represented on the depression component. The correlation of immobility during the test but not the training session with local activity on the EPM may point to different underlying forms of anxiety (Carola et al., 2002). This may be supported by the results of other studies in which a positive correlation between the time spent in the open-arm of the elevated plus maze and the duration of immobility in the forced swim test (Estanislau et al., 2011) or a negative correlation between FS-immobility and open-field locomotor activity (Ho et al., 2002) in rats has been found. However, a possible common underlying behavioral pattern is difficult to identify. Generally, the PCA combines the within-test variables and discriminates the between-test variables more clearly than the regression analysis.

Sprague-Dawley is an outbred rat strain with broader genetic variability that may reflect the situation in a human population more realistically, and most of the studies in rodents are conducted as an animal model for mechanisms in human populations. However, also in inbred strains with a narrow genetic variance genetic similarity can only explain a part of behavioral variability in home cage behavior (Loos et al., 2014) and behavioral tests (Vorhees, 1983; Lahmame and Armario, 1996; van der Staay and Blokland, 1996) not only in rodents but also in inbred human populations (Fareed and Afzal, 2014) such that individuality in behavior and trait behavior is still a considerable factor influencing the variance in experimental results (Sequeira-Cordero et al., 2014; Shumake et al., 2014). Further, different early pre-and postnatal experiences and environmental complexity support individual behavior, physiology, and molecular processes during adulthood (Oitzl et al., 2000; Braun and Champagne, 2014; Sarro et al., 2014). For these reasons it is difficult to generalize between different laboratories but individuality should be estimated for each local population of animals and may then provide more reliable results in animal models of cognitive diseases and individual vulnerability (Koolhaas et al., 2010). Environmental standardization of test procedures has been found to be more the cause than the remedy of low reproducibility of behavioral experimental outcomes between laboratories (Richter et al., 2009). The main reason is that the local conditions are stressed and the validity to other laboratories impaired. The study shows that considering individual factor loadings in multivariate analyses supports the characterization of individuality. A characterization of local populations as suggested here may improve the external validity.

## Author contributions

DF and YA performed the experiments; EE and HH wrote the manuscript; GL designed the experiments and wrote the manuscript; VK designed the experiments, performed the analyses, and wrote the manuscript.

### Conflict of interest statement

The authors declare that the research was conducted in the absence of any commercial or financial relationships that could be construed as a potential conflict of interest.
