# Supplementary material for: Individual Differences in Male Rats in a Behavioral Test Battery: A Multivariate Statistical Approach
Source: Front Behav Neurosci. 2017 Feb 17;11:26. doi: 10.3389/fnbeh.2017.00026 (PMC5314104; doi:10.3389/fnbeh.2017.00026)
Supplement: Supplementary file 1 [file Table1.PDF]

	Trial 1	Trial 6	Trial 10	OFRDR	OFlocal	OF-veloc	RRtime
Number of values	162	162	162	160	160	160	147
Minimum	0	0	0	0.002114	0.68	0	8.333
25% Percentile	0	0	0	0.04729	10.65	0.01	26.33
Median	0.1429	0	0	0.07018	15.67	0.01	32.67
75% Percentile	0.4	0.5	0.7569	0.1112	20.38	0.01	38
Maximum	1	1	1	0.3927	37.17	0.04	72
Mean	0.2155	0.2375	0.3178	0.08566	15.87	0.01144	33.14
Std. Deviation	0.249	0.3597	0.4146	0.06247	7.563	0.007341	10.18
Std. Error	0.01957	0.02826	0.03258	0.004938	0.5979	0.0005804	0.8399
Lower 95% CI of mean	0.1769	0.1817	0.2534	0.0759	14.69	0.01029	31.48
Upper 95% CI of mean	0.2542	0.2933	0.3821	0.09541	17.05	0.01258	34.8
Coefficient of variation	115.54%	151.41%	130.49%	72.93%	47.65%	64.19%	30.73%

	FSIMTR	FSIMT	EPMRCO	EPMlocal	EPMOE	EPMRDOC
Number of values	159	159	160	160	160	157
Minimum	0.4983	0.54	0	25.99	0	0
25% Percentile	0.96	1.417	2.282	62.34	7	3.175
Median	1.21	2.13	4.467	66.16	13	5.251
75% Percentile	1.458	2.88	9.862	68.93	18	11.55
Maximum	3.215	19.73	376.2	78.62	30	93.94
Mean	1.26	2.366	14.18	63.29	12.89	10.81
Std. Deviation	0.4677	1.75	37.54	10.07	6.641	14.85
Std. Error	0.03709	0.1388	2.968	0.7965	0.525	1.185
Lower 95% CI of mean	1.187	2.092	8.319	61.72	11.86	8.473
Upper 95% CI of mean	1.333	2.64	20.04	64.87	13.93	13.15
Coefficient of variation	37.12%	73.98%	264.75%	15.92%	51.51%	137.30%

Supplementary Table 1. Statistical measures and numbers of valid cases for each behavioral variable over the entire cohort.

HB-trial 1,6,10: Holeboard reference memory index for trial 1, 6 and 10; respectively. OFRDR, -local, -veloc: Ratio between distance travelled and resting, local movement and mean velocity in the open field; respectively. RR-time: time to be on the rotarod. FSIMTR, -T: time in percent spent immobile in the forced swim task during training and test session, respectively. EPMRCO, -local, -OE, -RDOC: Ratio between time spent in open and closed arms, local movement, number of entries in open arms, ratio between distance travelled in open and closed arms on the elevated plus-maze; respectively.
