# Supplementary material for: Individual Differences in Male Rats in a Behavioral Test Battery: A Multivariate Statistical Approach
Source: Front Behav Neurosci. 2017 Feb 17;11:26. doi: 10.3389/fnbeh.2017.00026 (PMC5314104; doi:10.3389/fnbeh.2017.00026)
Supplement: Supplementary file 2 [file Image1.PDF]

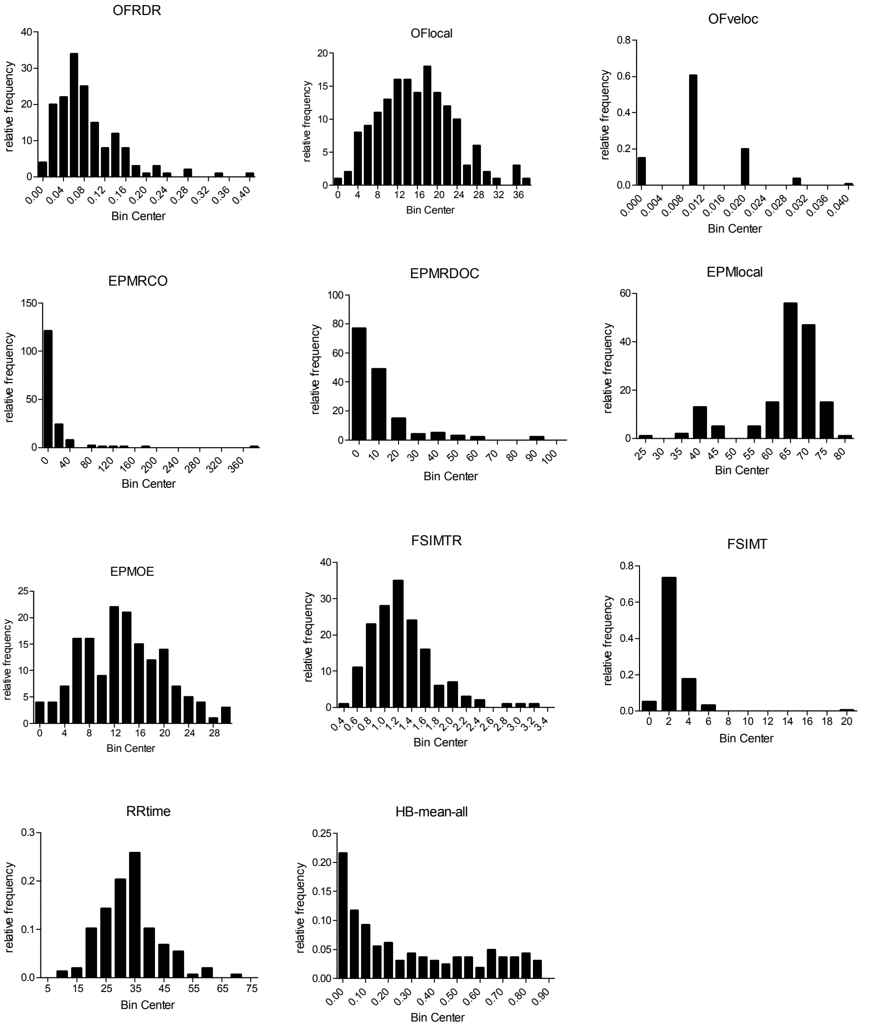

Supplementary figure 1 . Frequency distribution of raw data across the entire population for different behavioral variables.
OFRDR, -local, -veloc: Ratio between distance travelled and resting, local movement and mean velocity in the open field; respectively. EPMRCO, -local, -OE, -RDOC: Ratio between time spent in open and closed arms, local movement, number of entries in open arms, ratio between distance travelled in open and closed arms on the elevated plus-maze; respectively. FSIMTR, -T: time in percent spent immobile in the forced swim task during training and test session, respectively. RR-time: time to be on the rotarod. HB-all: Mean Holeboard reference memory index for all trials.
